# Tau Deletion Prevents Cognitive Impairment and Mitochondrial Dysfunction Age Associated by a Mechanism Dependent on Cyclophilin-D

**DOI:** 10.3389/fnins.2020.586710

**Published:** 2021-02-10

**Authors:** Claudia Jara, Waldo Cerpa, Cheril Tapia-Rojas, Rodrigo A. Quintanilla

**Affiliations:** ^1^Laboratory of Neurodegenerative Diseases, Universidad Autónoma de Chile, Santiago, Chile; ^2^Laboratory of Neurobiology of Aging, Centro de Biología Celular y Biomedicina (CEBICEM), Universidad San Sebastián, Santiago, Chile; ^3^Laboratorio de Función y Patología Neuronal, Departamento de Biología Celular y Molecular, Facultad de Ciencias Biológicas, Pontificia Universidad Católica de Chile, Santiago, Chile

**Keywords:** tau, Alzheimer’s disease, aging, mitochondria, cyclophilin-D, hippocampus, memory

## Abstract

Aging is an irreversible process and the primary risk factor for the development of neurodegenerative diseases, such as Alzheimer’s disease (AD). Mitochondrial impairment is a process that generates oxidative damage and ATP deficit; both factors are important in the memory decline showed during normal aging and AD. Tau is a microtubule-associated protein, with a strong influence on both the morphology and physiology of neurons. In AD, tau protein undergoes post-translational modifications, which could play a relevant role in the onset and progression of this disease. Also, these abnormal forms of tau could be present during the physiological aging that could be related to memory impairment present during this stage. We previously showed that tau ablation improves mitochondrial function and cognitive abilities in young wild-type mice. However, the possible contribution of tau during aging that could predispose to the development of AD is unclear. Here, we show that tau deletion prevents cognitive impairment and improves mitochondrial function during normal aging as indicated by a reduction in oxidative damage and increased ATP production. Notably, we observed a decrease in cyclophilin-D (CypD) levels in aged tau−/− mice, resulting in increased calcium buffering and reduced mitochondrial permeability transition pore (mPTP) opening. The mPTP is a mitochondrial structure, whose opening is dependent on CypD expression, and new evidence suggests that this could play an essential role in the neurodegenerative process showed during AD. In contrast, hippocampal CypD overexpression in aged tau−/− mice impairs mitochondrial function evidenced by an ATP deficit, increased mPTP opening, and memory loss; all effects were observed in the AD pathology. Our results indicate that the absence of tau prevents age-associated cognitive impairment by maintaining mitochondrial function and reducing mPTP opening through a CypD-dependent mechanism. These findings are novel and represent an important advance in the study of how tau contributes to the cognitive and mitochondrial failure present during aging and AD in the brain.

## Introduction

Aging is a biological process associated with progressively accumulating damage in the organism and also is the principal risk factor for several neurodegeneration diseases, including Alzheimer’s disease (AD) (Stauch et al., 2014; Xia et al., 2018). Concerning brain function, aging leads to the deterioration of cognitive capacities as a consequence of synaptic alterations (Wyss-Coray, 2016) and loss of neurons in the hippocampus (Swerdlow, 2011; Lopez-Otin et al., 2013). Tau protein regulates the dynamics of microtubules (Tapia-Rojas et al., 2019a). However, post-translational tau modifications dissociate it from the microtubules, leading to the formation of aggregates (Wang et al., 2010; Wyss-Coray, 2016), and inducing alterations in synaptic and cognitive functions (Avila et al., 2013; Zuo et al., 2016). Nevertheless, misfolded tau is detected in the brain of approximately one-third of elderly people without dementia (Elobeid et al., 2016), which may contribute to the neuronal alterations observed in aging. Additionally, these pathological forms of tau play a relevant role in the onset and progression of AD (Xia et al., 2018), but the exact mechanism leading to neural toxicity is unclear. In AD brains, tau is ∼three to fourfold more hyperphosphorylated than in normal conditions, forming paired helical filaments (PHF), and intraneuronal neurofibrillary tangles (NFT) (Iqbal et al., 2010).

Mitochondria are organelles required for ATP generation, calcium regulation, and maintenance of redox balance (Sebastian et al., 2017; Perez et al., 2018a). Mitochondrial dysfunction has been proposed as the common denominator connecting aging and the pathogenesis of AD (Du et al., 2012; Jara et al., 2019; Wu et al., 2019). Indeed, mitochondrial and synaptic dysfunction are the early characteristics of AD (Perez et al., 2018a). The loss of mitochondrial function leads to increased production of reactive oxygen species (ROS), decreased ATP formation (Sebastian et al., 2017), and reduced calcium-buffering capacity (Rottenberg and Hoek, 2017). Interestingly, these events could be associated with mitochondrial permeability transition pore (mPTP) opening (Perez and Quintanilla, 2017), which plays a relevant role in aging and AD (Du et al., 2011). Importantly, the formation and opening of the mPTP is enhanced by cyclophilin-D (CypD) expression (Du et al., 2008; Gauba et al., 2017; Hurst et al., 2017; Panel et al., 2018). Also, relevant studies have shown increased CypD levels in AD brain samples and mice models (Du et al., 2008). More importantly, CypD (-/-) mice crossing with APP/PS1 AD mice model that presented neurotoxicity and memory impairment prevented mitochondrial dysfunction, synaptic impairment, and cognitive decline indicating an important contribution to CypD in neurodegenerative changes shown in AD (Du et al., 2011).

Interestingly, a novel association between mitochondrial dysfunction and tau pathology contributing to AD pathology has been shown (Quintanilla et al., 2012, 2014; Perez et al., 2018a). We showed that the expression of pathological forms of tau (phosphorylated and truncated) promote mitochondrial depolarization, mitochondrial fragmentation, and oxidative stress, compromising mitochondrial function (Quintanilla et al., 2009, 2014; Perez et al., 2018b). More importantly, in a recent study, we showed that tau ablation enhanced cognition and improved mitochondrial function in the hippocampi of young mice compared to wild-type animals (Jara et al., 2018). However, the contribution of tau and mitochondria to the normal aging process is unclear and controversial. Some reports have shown that tau knockdown impairs brain capacity, including motor and cognitive function in the adult brain (Lei et al., 2012; Velazquez et al., 2018). In contrast, Morris et al. (2013) showed that the absence of tau did not affect cognition performance in aged mice. Considering these discrepancies, we investigated the effects of tau absence on behavioral impairment and mitochondrial function during aging, using 18-month-old wild-type (WT) and tau-knockout (tau−/−) mice. We observed that tau−/− mice maintained their cognitive capacities during aging, including hippocampal memory and social behavior. Besides, we observed a reduction in oxidative damage and higher ATP levels in the aged tau−/− animals. Interestingly, we observed reduced levels of CypD and lower sensitivity to calcium overload in hippocampal mitochondria from the aged tau−/− mice, suggesting a reduced activity of mPTP. To corroborate that CypD deficiency is involved in the mitochondrial and cognitive improvement observed in aged tau−/− mice, we overexpressed CypD in the hippocampus of these mice using a lentiviral vector (Li et al., 2012; Parr-Brownlie et al., 2015). Notably, CypD overexpression was sufficient to impair mitochondrial calcium buffer capacity and to reduce ATP production in aged tau−/− mice. Most importantly, the cognitive abilities observed in the aged tau−/− mice were significantly reduced. Thus, genetic reduction of tau preserves mitochondrial bioenergetics and cognitive abilities during aging by a mechanism that involves CypD and possibly the mPTP opening in the hippocampus.

## Materials and Methods

### Animals

Wild-type (WT, C57BL/6J background) and homozygous tau-knockout (tau−/−) mice were obtained from the Jackson Laboratory (B6.129-Mapttm1Hnd/J Bar Harbor, ME, Stock N°007251). WT C57BL/6J mice were used as litter control, considering the control suggestions by The Jackson Laboratory and also that tau KO strain have this genetics background. The animals were handled according to the guidelines of the National Institute of Health (NIH, Baltimore, MD). They were maintained at the Bioterio Central of Universidad Autónoma de Chile. All mice were housed at 23°C and on a 12-h light/dark cycle with food and water *ad libitum*. Experimental procedures were approved by the Bioethical and Biosafety Committee of the Universidad Autónoma de Chile. All studies were conducted on wild type (WT) and tau KO (tau−/−) littermates. A total of nine WT and seven tau−/− mice, 18 months old were used in the cognitive tests. For the biochemical studies, we used additional (*n* = 3) WT and tau−/− mice. For mitochondrial analysis, we used a separate cohort with *n* = 4 mice per group. All groups include female and male animals, with differences observed between sex.

### Reagents and Antibodies

The primary antibodies used were as follows: Anti-β-tubulin (sc-9104, Santa Cruz Biotechnology, Inc., 1:2,000), anti-Total OXPHOS Human WB Antibody Cocktail (ab110411, Abcam, Inc. 1:2,000), anti-COX IV (11967S, Cell Signaling, 1:1,000), anti-β-actin (sc-47778, Santa Cruz Biotechnology, Inc., 1:1,000), anti-Opa1 (PA1-16991, Thermo Fisher Scientific, 1:1,000), anti-Cyclophilin D (sc-376061, Santa Cruz Biotechnology, Inc., 1:1,000), anti-ANT (Santa Cruz biotechnology; 1:1,000), anti-Mfn1 (sc-50330, Santa Cruz Biotechnology, Inc. 1:1,000), anti-Mfn2 (sc-50331, Santa Cruz Biotechnology, Inc., 1:1,000), anti-phospho-DRP1 (Ser616) (4494, Cell Signaling, 1:1,000), anti-DRP1 (sc-271583, Santa Cruz Biotechnology, Inc., 1:1,000), anti-nitrotyrosine (141682, US Biological, Life Sciences, 1:500), and anti-4HNE (H6275-02, US Biological, Life Sciences, 1:1,000). The fluorescent dyes used were as follows: MitoTracker^TM^ Red CM-H2Xros (M7513, Thermo Fisher Scientific), MitoTracker^TM^ Green FM (M7514, Thermo Fisher Scientific), and VECTASHIELD Mounting Medium with DAPI (H1200, Vector Laboratories, Inc.).

### Behavioral Tests

All behavioral tests were monitored using an automatic tracking system (ANY-maze Behavioral tracking software, United Kingdom).

#### Novel Object Recognition (NOR) Test

NOR tests were performed in a 38 × 38 × 32 cm acrylic box and were performed according to Jara et al. (2018). The animals were habituated in the box for 2 consecutive days, without any object. During testing, each animal was placed in the box containing two identical objects (old objects) for 10 min. Then, the box and objects were cleaned (50% methanol). After 2 h, the animal was exposed to one of the old objects and a new object of different shapes and colors. The recognition index was calculated as the time spent by the mouse exploring the new object divided by the time spent exploring both objects.

#### Barnes Maze Test

Barnes maze test was performed according to Olesen et al. (2020). The paradigm consists of an elevated circular platform, with 20 equally spaced holes along the perimeter. The animals must learn to escape from the open platform surface to a small chamber located under the platform, guided by visual–spatial cues. For reinforcement, we used aversive white noise. During the acquisition phase, the mouse was placed in a cylindrical black. After 5 s, white noise was switched on, and the mouse explored. The trial ended when the mouse entered the escape chamber or after 3 min of exploration. When the mouse entered the escape chamber, the white noise was turned off. If the mouse did not achieve this criterion, it was guided to the escape chamber. This protocol was repeated thrice on day 1 and twice on day 2. Forty-eight hours after training, the time spent on the mouse until finding the escape chamber was evaluated.

#### Social Interaction Test

For this task, a previously described protocol was used (Jara et al., 2018). Briefly, the mice were habituated in a three-chamber box (each chamber was 20 × 40 × 22 cm) for 10 min. Subsequently, one object and one unknown mouse were placed inside a cage and were presented to the experimental mouse, one in each lateral chamber. Each mouse was positioned in the central chamber and was allowed to explore the cage for 10 min. In the final part of the test, an unknown mouse replaced the previous object. The experimental mouse was allowed to explore for 10 min.

#### Object-Based Attention Test

For this task, a previously described protocol was used (Jara et al., 2018). Briefly, the test was performed in a rectangular apparatus, containing two chambers, which included the exploration chamber (40 × 40 × 22 cm) and the test chamber (40 × 20 × 22 cm). First, the animals were exposed to the habituation phase, a session of 10 min exploring both empty chambers. Later, during the acquisition phase, the mice were subjected to a 3-min session exploring five objects (1, 2, 3, 4, and 5) distributed within the chamber. Finally, in the retention phase, immediately after the acquisition phase (<15 s), an old object (used in the acquisition phase) was placed in its original position, and a sixth novel object was placed in the test chamber. The mice explore both objects for 3 min. A recognition index is calculated as (T6 × 100)/(T2 + T6), where T2 and T6 are the time duration spent by each mouse with objects 2 and 6, respectively.

### Total RNA Extraction

Total RNA was isolated from 100 mg of tissue using the TRIzol reagent (Life Technologies, Thermo Fisher Scientific, United States) following the manufacturer’s instructions. Residual DNA was removed with RNase free-DNase I, Amplification Grade (Invitrogen, Thermo Fisher Scientific). RNA yield and purity were determined by a TECAN plate reader (Infinite 200 PRO series).

### Reverse Transcription for cDNA Synthesis

One microgram of RNA was subjected to reverse transcription using ImProm-II Reverse Transcription System (Promega) by the manufacturer’s protocol. For qPCR analysis, the cDNA sample was diluted 10× in nuclease-free water.

### Real-Time PCR

The real-time PCR reaction was performed in triplicates in the LightCycler 96 System (Roche Diagnostics GmbH, Roche Applied Science, Mannheim, Germany) using KAPA SYBR FAST qPCR Master Mix (2×) in a final reaction volume of 10 μl. Amplification conditions consisted of an initial hot start at 95°C for 10 min followed by amplification for 40 cycles (95°C for 15 s, 60°C for 20 s, and 72°C for 20 s). Melting curve analysis was performed immediately after amplification from 55 to 95°C. Values were normalized to 18S expression levels using the ΔCT method.

**Table:** 

Gene	Forward primer	Reverse primer
CypD	5′-AGGAGATAGCCCCAGGAGAT-3′	5′-TTGCATACACGGCCTTCTCTT-3′

### Western Blot

The hippocampi of WT and tau−/− mice were dissected and immediately processed (Jara et al., 2018). Briefly, the hippocampal tissues were homogenized in RIPA buffer (10 mM Tris-Cl, pH 7.4, EDTA 5 mM, 1% NP-40, 1% sodium deoxycholate, and 1% SDS) supplemented with a protease/phosphatase inhibitors mixture (25 mM NaF, 100 mM Na_3_VO_4_, and 30 μM Na_4_P_2_O_7_). The protein samples were centrifuged at 14,000 rpm for 15 min at 4°C. The protein concentrations were determined using the BCA Protein Assay Kit (Pierce, Thermo Fisher Scientific, United States). The samples were resolved by SDS-PAGE, followed by immunoblotting on PVDF membranes.

### Hippocampal Slices and Staining With Mitochondrial Fluorescent Dyes

Coronal 20-μm-thick slices of unfixed tissue were obtained from the brain of WT and tau−/− mice and stained (Jara et al., 2018; Tapia-Rojas et al., 2019b; Torres et al., 2020). Slices were mounted on slides and incubated with MitoTraker Green FM (mitochondrial mass) plus MitoTraker Red CM-H2Xros (mitochondrial membrane potential), in KRH buffer for 45 min at 37°C. After incubation, the slices were washed three times for 5 min in PBS and mounted with DAPI mounting medium. Images were acquired with a fluorescence microscope (LX 6000X, Leica, Germany).

### Measurement of ATP Concentration

ATP concentration was measured in the hippocampal lysates using a luciferin/luciferase bioluminescence assay kit (ATP determination kit #A22066, Molecular Probes, Thermo Fisher Scientific, United States), as previously described (Jara et al., 2018). The amount of ATP in each sample was calculated from standard curves and normalized to the total protein concentration.

### Isolation of Hippocampal Mitochondria

Hippocampal mitochondria were isolated as previously described (Jara et al., 2018; Carreras-Sureda et al., 2019). Briefly, four mice per group (WT and tau−/−) were euthanized, and the hippocampi were rapidly removed and suspended in MSH buffer (230 mM mannitol, 70 mM sucrose, 5 mM HEPES, pH 7.4) supplemented with 1 mM EDTA and protease inhibitor cocktail. Homogenates were centrifuged at 600 g for 10 min at 4°C to discard nuclei and cell debris. The supernatant was centrifuged at 8,000 g for 10 min; the mitochondrial pellet was washed twice in MSH without EDTA.

### Evaluation of Mitochondrial Calcium Buffering Capacity

Mitochondrial swelling was measured by monitoring absorbance decline at 540 nm in a fresh mitochondrial fraction (Karadayian et al., 2015; Olesen et al., 2020). Intact mitochondria scatter light at 540-nm wavelength. The prolonged mPTP opening provokes the swelling of mitochondria, which reduces the absorbance. Isolated mitochondria were resuspended in MSH buffer containing 5 mM malate, 5 mM glutamate, 1 mM phosphate, and 2 mM MgCl_2._ Mitochondrial samples (0.5 mg/ml of total protein) were exposed to different calcium concentrations to generate calcium overload, which induces mitochondrial swelling associated with mPTP opening.

### Surgical Procedures

Animals received bilateral intrahippocampal administration of Lenti ORF particles (GFP-tagged)-mouse peptidylprolyl isomerase D (Ppid; cyclophilin D) (Type: Human Tagged ORF Clone Lentiviral Particle; Tag: mGFP; Vector: pLenti-C-mGFP, #RC223397L2V, Origene) by stereotaxic injection (Vargas et al., 2014). A total of *n* = 5 mice were used per group. We used Sham injection in WT and control tau KO mice. Tau−/− mice were anesthetized using isoflurane and placed in a stereotaxic frame (Stoelting, United States). The skull was exposed for several millimeters anterior and posterior to the bregma. Boreholes were made above the left and right hippocampal CA1 (coordinates: 2.46 mm anterior to the bregma, 1.0 mm lateral, 1.5 mm relative to dura mater). One microliter of the lentiviral vector was injected (10^8^ TU/ml). Three weeks after infection, the animals were subjected to cognitive tests and euthanized immediately after for biochemical analysis.

### Statistical Analysis

The data are expressed as the mean ± standard error of the mean (S.E.M.). The data was analyzed using the Student’s *t*-test with Dunnett’s *post-hoc* test or one-way ANOVA followed by Bonferroni’s *post-hoc* test. A value of *p* < 0.05 is statistically significant. All statistical analyses were performed using Prism software (GraphPad Software Inc.).

## Results

### The Absence of Tau Prevents the Impairment of Hippocampus-Dependent Memory During Aging

**FIGURE 1:**
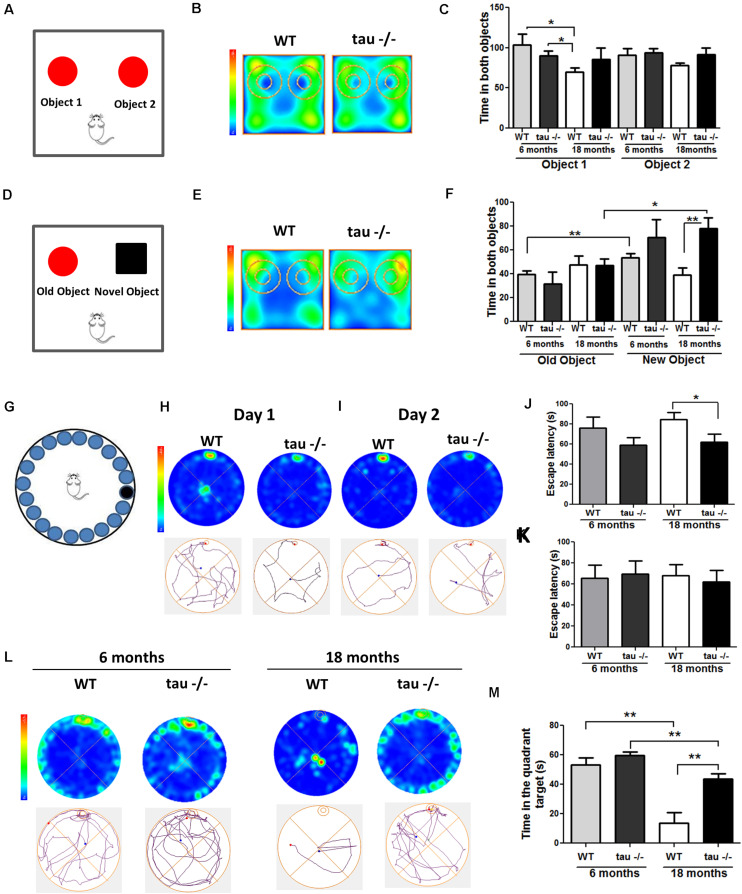
The absence of tau prevents the loss of hippocampal-dependent memory during aging. **(A)** Scheme of the novel object recognition (NOR) test, familiarization phase. **(B)** Heat maps of the aged WT and tau−/− groups in the NOR familiarization phase. **(C)** Graph of exploration time for both objects 1 and 2, during the familiarization phase. **(D)** Scheme of NOR testing phase; 2 h after the familiarization phase, the mice explored a familiar object and a novel object. **(E)** Heat maps of the aged WT and tau−/− groups during the NOR testing phase. **(F)** Graph of exploration time of both old and novel objects, during the testing phase. **(G)** Representation of the Barnes maze. **(H,I)** Heat maps and tracks of aged WT and tau−−/−− mice in the Barnes maze, during training days 1 and 2. **(J,K)** Escape latency (time each mouse spent to find the escape chamber) on training days 1 and 2. **(L)** Heat maps of aged WT and tau−/− mice and representative tracks until the finding of the escape chamber. **(M)** Graph of the time in the escape chamber quadrant. *p < 0.05, **p < 0.01; mean ± S.E.M.

The hippocampus is susceptible to aging (Bartsch and Wulff, 2015). Neurobiological alterations in the aged hippocampus include oxidative damage and altered communication among neurons (Bettio et al., 2017; Jara et al., 2019). These events result in impaired hippocampal function, affecting learning and memory (Bettio et al., 2017; Jara et al., 2019). Pathological forms of tau have been linked to memory-related disorders, such as AD (Amadoro et al., 2010; Pooler et al., 2013). However, it is unclear if tau contributes to memory loss during the physiological aging process. To determine the impact of the absence of tau on hippocampal memory impairment during aging, 6- and 18-month-old (mo) WT and tau−/− mice were subjected to behavioral tests, including the Novel Object Recognition (NOR) test, which evaluates recognition memory, and the Barnes maze test, which is a spatial memory task (Broadbent et al., 2004). For the NOR test, mice were subjected to the familiarization phase. During this stage, each animal explored the chamber containing two identical objects, for 10 min (Figure 1A). The behaviors of the 18-month-old WT and tau−/− mice during the familiarization phase are shown in the heat maps in Figure 1B. We observed that the 18-month WT mice explored the chamber with the two objects for less time than both the aged tau−/− and the 6-month mice of both genotypes (Figure 1C). These observations suggested that aged WT mice had reduced explorative abilities, whereas tau−/− mice maintained their capacity. Two hours later, the recognition phase was performed. During this stage, the mice explored the chamber containing a familiar object and a novel object for 5 min (Figure 1D). Figure 1E presents the heat maps for the performance of the 18-month mice during this phase. The aged tau−/− mice showed preference for the novel object, in contrast to the aged WT mice. Quantitative analysis revealed that during this phase, the 18-month tau−/− animals spent significantly more time exploring the chamber for the novel object compared to the WT mice of the same age (Figure 1F). In addition, the behavior of the aged tau−/− mice was similar to that of the 6-month tau−/− mice, suggesting that tau ablation preserves the recognition memory that is normally affected during aging.

**FIGURE 2:**
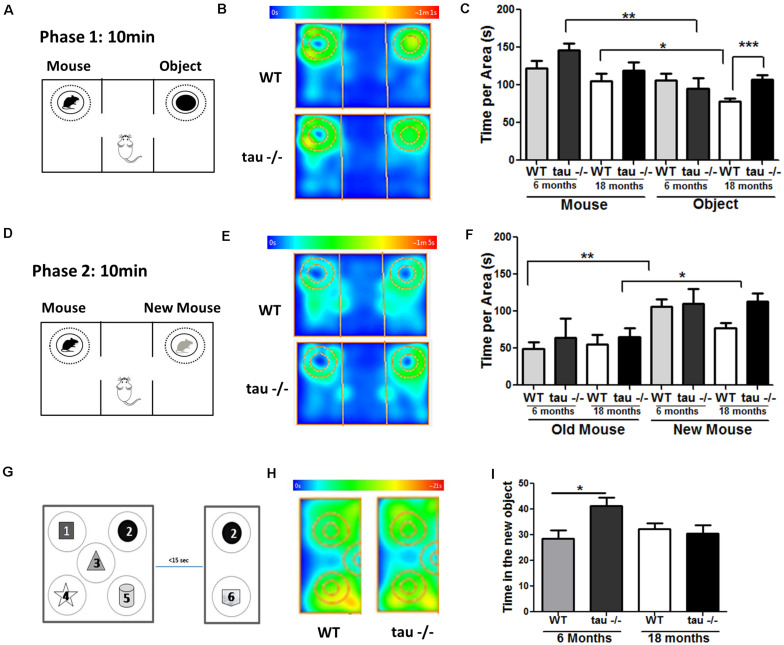
Tau−/− mice maintain their social abilities during aging. **(A)** Scheme of phase 1 of the social interaction test. **(B)** Heat maps of aged WT and tau−/− mice during phase 1 of the social interaction test. **(C)** Graph of the exploration time of both the mouse and the object. **(D)** Scheme of phase 2 of the social interaction test. **(E)** Heat maps of aged WT and tau−/− mice during phase 2 of the social interaction test. **(F)** Graph of the exploration time of both the old and new mice. **(G)** Scheme of the object-based attention test. Mice were exposed to five objects (1–5) for 3 min (exploration phase). After a 15-s interval, the mice were exposed to one old (2) and one novel object (6) for 3 min. **(H)** Heat maps showing data for the aged mice in the test. **(I)** Graph of time spent for the exploration of the new object. *p < 0.05, **p < 0.01, ***p < 0.001; mean ± S.E.M.

Next, we evaluated the impact of tau deletion on spatial learning and memory, another type of hippocampus-dependent memory that is reduced during aging (Rosenbaum et al., 2012). For this purpose, we performed the Barnes maze test (Figure 1G). Figures 1H,I show the heat maps of 18-month WT and tau−/− mice on training day 1 (Figure 1H) and 2 (Figure 1I). On each day, we measured the time that the mice spent finding the escape chamber (escape latency). Aged tau−/− mice exhibited reduced escape latency compared to the aged WT mice (Figure 1J). These findings suggest that the aged tau−/− mice learn faster than the aged WT mice. However, during the second training day, all experimental groups were located in the escape chamber with a similar latency (Figure 1K). Finally, 2 days after training, the trial was performed in the absence of an escape chamber. The behavior of each experimental group is shown in the heat maps and the representative tracks in Figure 1L. We evaluated the time spent in the quadrant where the escape chamber was previously located, and interestingly, we observed that the aged tau−/− mice remembered the escape zone, as did the animals belonging to both 6-month groups. In contrast, the 18-month WT mice did not remember the escape chamber location (Figure 1M). Therefore, these studies indicate that the absence of tau prevented the loss of spatial memory observed in the aged WT animals. Thus, tau deletion preserves recognition and spatial memory that are normally impaired during aging.

### Tau−/− Mice Maintain Their Social Abilities During Aging

During aging, decreased social contact has been reported (Shoji et al., 2016). Sociability and social memory are processed by the prefrontal cortex, hippocampus, hypothalamus, and amygdala (Bicks et al., 2015). In aging, a reduced preference for novel conspecific individuals occurs (Charles and Carstensen, 2010; Smith et al., 2018). To evaluate whether tau deletion modified social abilities during aging, we performed a social interaction test (Figure 2; Jara et al., 2018). During the first phase, the mice explored a chamber containing a mouse and an object for 10 min (Figure 2A). Figure 2B shows heat maps of the 18-month WT and tau−/− mice. We observed that all groups showed a preference for exploring the unknown mouse compared with the unknown object, suggesting a similar ability to socialize (Figure 2C). Subsequently, the object was replaced by a new unknown mouse, and the experimental were animals to explore the chamber for another 10 min (Figure 2D). The heat maps showed that aged tau−/− mice spent more time with the new mouse compared with the aged WT mice (Figure 2E). We found that both 6-month groups preferred exploring the new mouse, in contrast to aged WT mice that spent similar time exploring the known and unknown mice (Figure 2F). Interestingly, this analysis also revealed that aged tau−/− mice also preferred exploring the new mouse (Figure 2F) indicating that the absence of tau maintains social recognition memory during aging.

Finally, we performed a recognition-based attention test to determine if aging affects the attentive behavior in WT and tau−/− mice, since variations in this behavior could be responsible for the changes detected in memory and social abilities (Chun and Turk-Browne, 2007). For this test, five different objects were placed in the chamber, as indicated in Figure 2G. The mice explored the chamber containing the objects for 3 min, and 15 s later, the chamber size was reduced, an old object remained in the chamber (object 2), and a new object was added (object 6). Figure 2H showed heat maps of the aged WT and tau−/− mice during the test phase. We found that the aged WT and tau−/− mice spent a similar time exploring the new object (Figure 2I). Therefore, our results indicated that 18-month WT and tau−/− mice did not show differences in their attention capacity during aging. Thus, the changes observed between WT and tau−/− mice could be related to memory loss occurring in the aged WT mice that are prevented by the absence of tau.

### Tau Absence Prevents Mitochondrial Bioenergetics Failure Observed During Aging

**FIGURE 3:**
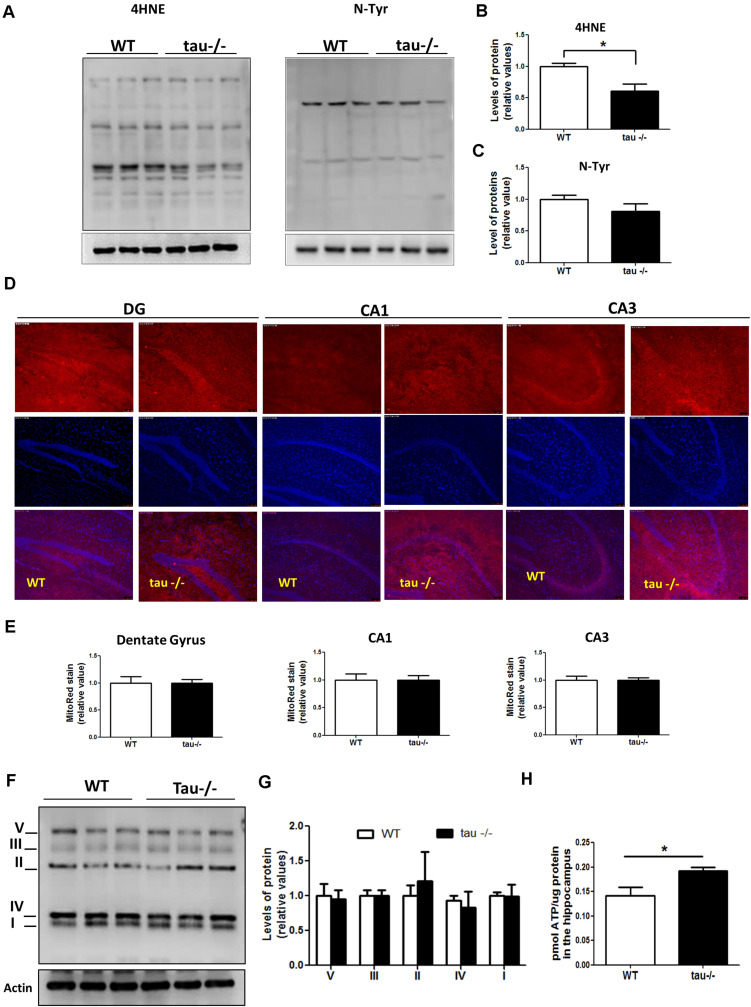
Tau absence prevents mitochondrial bioenergetics impairment observed during aging. **(A)** Western blot of hippocampal lysates from aged WT and tau−/− mice for the measurement of oxidative damage with antibodies against 4HNE and n-Tyr. **(B,C)** Densitometric analysis of 4HNE **(B)** and n-Tyr **(C)** Western blots. **(D)** Representative images of unfixed hippocampal slices from aged WT and tau−/− mice, stained with MitoTracker Red CM-H2Xros. **(E)** Quantitative analysis of MitoTracker Red CM-H2Xros fluorescence intensity in the DG, CA1, and CA3 hippocampal regions. **(F)** Western blot and **(G)** densitometric analysis for mitochondrial OXPHOS complexes I–V in whole hippocampal extracts from aged WT and tau−/− mice. **(H)** ATP concentrations in the hippocampus of aged WT and tau−/− mice, expressed as pmol of ATP/μg of total protein. *p < 0.05; mean ± S.E.M. 4HNE, 4-hydroxynonenal; DG, dentate gyrus; n-Tyr, nitrotyrosine; OXPHOS, oxidative phosphorylation.

Mitochondrial dysfunction contributes to aging by increasing the production of ROS and promoting deficits in the bioenergetic processes (Chistiakov et al., 2014; Jara et al., 2019). Oxidative damage and aging are strongly connected (Rottenberg and Hoek, 2017). To determine if the absence of tau has a significant effect on the oxidative damage occurring during aging, we dissected the hippocampi of 18-month WT and tau−/− mice, and the samples were analyzed by Western blot. We evaluated the levels of oxidized proteins using an anti-HNE antibody that recognizes the stably formed HNE-protein adducts (products of lipid peroxidation) (Bettio et al., 2017), and anti-nitrotyrosine antibody that detects protein modifications involving nitrotyrosine (Jara et al., 2018). Our results showed that the levels of lipid peroxidation products were significantly reduced in the hippocampi of the aged tau−/− mice in comparison to the WT mice of the same age, as indicated by densitometry analysis (Figures 3A,B), whereas nitrotyrosine protein levels showed no significant differences between the two experimental groups (Figures 3A,C). These results show that the absence of tau reduced oxidative damage mediated by peroxidation in the aged tau−/− animals.

On the other hand, mitochondria are dynamic organelles that change their size depending on intracellular and extracellular signals (Sebastian et al., 2017). During aging, mitochondria are prone to gradual deterioration (Chistiakov et al., 2014). For this reason, we measured the mitochondrial mass in the hippocampi of aged WT and tau−/− mice and did not detect any difference between the two groups (Supplementary Figure 1A). Besides, mitochondria undergo continuous cycles of fusion/fission events, influencing their functionality (Sebastian et al., 2017). Therefore, we measured the levels of proteins involved in mitochondrial dynamics in both aged WT and tau−/− mice (Supplementary Figure 1C). Fusion events are controlled by the dynamin-related GTPases as well as mitofusins (Mfn1 and Mfn2) and optic atrophy 1 (OPA1) proteins, which induce the fusion of outer and inner mitochondrial membranes, respectively (Hood et al., 2018). In contrast, fission is mediated by dynamin-related protein 1 (Drp1), which is recruited to the outer membrane to constrict mitochondria and induce their division, a process that is mainly stimulated by Drp1 phosphorylation at Ser616 (Sebastian et al., 2017). We observed that both fission and fusion protein levels were not significantly different between the experimental groups (Supplementary Figure 1B). Therefore, the absence of tau did not affect the expression of the proteins that regulate mitochondrial dynamics during aging.

Mitochondria are the main producers of ATP in neurons, and during the aging process, they are particularly susceptible to damage (Schon and Manfredi, 2003; Jara et al., 2019). To determine whether the absence of tau could be beneficial for mitochondrial bioenergetics during aging, we incubated unfixed hippocampal slices of aged WT and tau−/− mice with the dye MitoTracker Red CMXH2Ros to measure the mitochondrial membrane potential. This dye detects functional mitochondria, and the fluorescence intensity is proportional to mitochondrial membrane potential (Figure 3D; Jara et al., 2018; Tapia-Rojas et al., 2019b). Figure 3D showed similar mitochondrial membrane potential levels in all regions of the hippocampus, including the dentate gyrus (DG), CA1, and CA3 of aged WT and tau−/− mice (Figure 3E). Also, we evaluated the protein levels of the mitochondrial respiratory complexes involved in oxidative phosphorylation (OXPHOS) using the antibody cocktail OXPHOS that contains a mix of antibodies specific for the five mitochondrial complexes (Jara et al., 2018). We observed that the two groups of mice had similar levels of all mitochondrial complexes (Figures 3F,G). Finally, the main mitochondrial function involves the production of ATP, and it is known that during aging, ATP formation is reduced in the hippocampus (Navarro and Boveris, 2010). Interestingly, when we evaluated ATP production, we observed that the 18-month tau−/− mice had significantly higher ATP levels compared to WT mice of the same age (Figure 3H). Therefore, our results indicate that the mitochondria in aged tau−/− mice maintain their bioenergetics capacity during aging.

### Aged Tau−/− Mice Exhibit Decreased Levels of CypD, Which Reduces mPTP Opening

**FIGURE 4:**
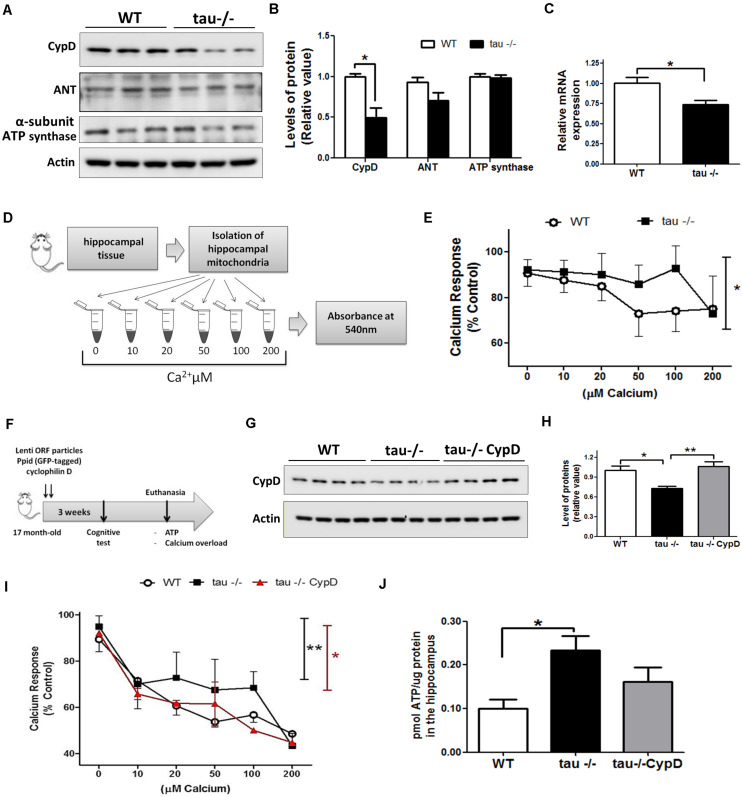
Reduced mitochondrial calcium buffering and improved bioenergetics in the hippocampi of aged tau−/− mice are reverted by overexpression of CypD. **(A)** Western blot of hippocampal lysates and **(B)** densitometric analysis of proteins that form the mPTP, including CypD, ANT, and ATP synthase in aged WT and tau−/− mice. **(C)** Relative mRNA expression of CypD in aged WT and tau−/− mice. **(D)** Representation of mitochondrial membrane swelling after calcium overload to determine mPTP opening in aged WT and tau−/− mice. **(E)** Response of isolated mitochondria after calcium overload. The decreased absorbance indicated mitochondrial swelling. **(F)** Representation of lentiviral vector transduction in tau−/− mice for the overexpression of CypD [Lenti ORF particles (GFP-tagged)-mouse peptidylprolyl isomerase D (Ppid; cyclophilin D) (#RC223397L2V. Origene)]. **(G)** Western blot of hippocampal lysates and **(H)** densitometric analysis of CypD protein in aged WT, tau−/− control, and tau−/− overexpressing CypD mice. **(I)** Graphical representation of the response of isolated mitochondria after calcium overload in aged WT, tau−/− control, and tau−/− CypD mice. **(J)** ATP concentrations measured in whole hippocampal extracts from aged WT, tau−/− and tau−/− CypD mice. ATP concentration is expressed as pmol of ATP/μg of total protein extract. *p < 0.05, **p < 0.01; mean ± S.E.M.

The levels of CypD are increased in the brain of WT mice during aging (Gauba et al., 2017). This is interesting because CypD is a fundamental protein for the formation of the mPTP (Du et al., 2008, 2011; Jara et al., 2019). To investigate whether tau ablation alters the levels of mPTP components, we performed Western blot analysis using hippocampal lysates from aged WT and tau−/− mice (Figure 4A). We observed that the levels of CypD were significantly reduced in the aged tau−/− mice compared with the WT mice of the same age, while the levels of adenine nucleotide translocase (ANT) and ATP synthase proteins, two recognized protein members of mPTP (Perez and Quintanilla, 2017), showed no significant differences between the two groups as observed by densitometry analysis (Figure 4B). Interestingly, we also observed decreased mRNA levels of CypD in the 18-month tau−/− mice (Figure 4C), suggesting decreased synthesis and expression of CypD.

Importantly, reduced CypD expression prevents mPTP opening in the brain and heart (Du et al., 2011; Hom et al., 2011). Considering that increased calcium concentrations in the mitochondria can induce mPTP opening (Baumgartner et al., 2009), we evaluated the sensitivity to calcium overload (mitochondrial calcium-buffering capacity) in enriched mitochondrial preparations from aged WT and tau−/− hippocampus. Fresh hippocampal mitochondria were exposed to different calcium concentrations (10, 20, 50, 100, and 200 μM CaCl_2_) as indicated in Figure 4D. To detect mitochondrial swelling as a consequence of mPTP opening by increasing calcium concentrations, we measured the absorbance changes at 540 nm (Karadayian et al., 2015). Figure 4E shows the calcium overload curves of both mouse groups, and interestingly, we observed that the aged tau−/− mice exhibited lower sensitivity to calcium overload compared to the aged WT mice (Figure 4E). These observations indicate that tau deletion could reduce premature mitochondrial mPTP opening induced by calcium overload.

### Hippocampal CypD Overexpression Reduced Mitochondrial Bioenergetics in the Aged Tau−/− Mice and Induced mPTP Opening

The expression levels of the main proteins involved in mPTP formation are modified during aging (Rottenberg and Hoek, 2017). CypD is increased in the brains of aged WT mice, and this could be responsible for mPTP opening (Gauba et al., 2017). To test the hypothesis that increased expression of CypD in WT mice is involved in mitochondrial dysfunction occurring in aged mice, we overexpressed CypD in aged tau−/− mice (17-month) by lentiviral transduction (Figure 4F). Intra-hippocampal viral administration was performed in both cerebral hemispheres. Three weeks after viral infection, the levels of CypD were measured. Similar to Figure 4B, we observed that the levels of CypD were significantly reduced in the aged tau−/− mice compared with the WT mice of the same age, while the levels of CypD in the tau−/− group infected with the lentiviral vector are significantly higher (Figures 4G,H). Then, we evaluated the mitochondrial sensitivity to calcium overload (Figure 4I). We observed that the aged tau−/− mice overexpressing CypD (tau−/− CypD) had increased calcium sensitivity compared to the aged tau−/− mice, which was similar to the aged WT mice (Figure 4I). Importantly, these results indicate that CypD overexpression is sufficient to reduce the mitochondrial calcium buffering capacity, similar to WT mice (Figure 4G). Finally, we measured the bioenergetics of mitochondria, by the evaluation of ATP production. Most importantly, we observed that the aged tau−/− CypD mice showed reduced ATP production compared with the aged tau−/− mice (Figure 4J). Interestingly, the ATP levels produced by the tau−/− CypD mice were similar to the levels observed in aged WT mice, suggesting that tau contributes to bioenergetics defects that occur during aging, by a mechanism that involves CypD. Thus, CypD overexpression in tau−/− mice replicated the mitochondrial defects observed in the aged WT mice and suggested that tau contributes to mitochondrial dysfunction in the hippocampus during the aging process.

### Overexpression of CypD Reduced the Improvement in Memory and Social Abilities Exhibited in the Aged Tau−/− Mice

**FIGURE 5:**
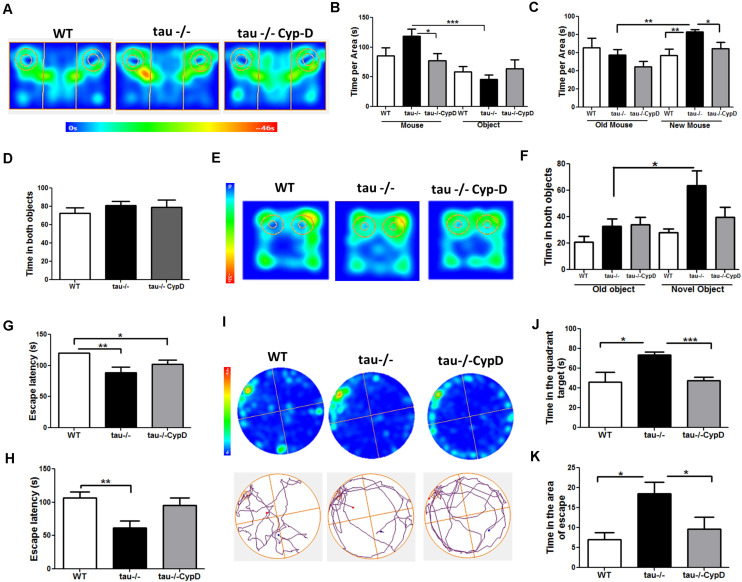
Tau−/− mice overexpressing CypD showed reduced social abilities and hippocampus-dependent memory similar to aged WT mice. **(A)** Heat maps of aged WT, tau−/− control, and tau−/− CypD mice during phase 1 of the social interaction test. **(B)** Graph of the exploration time of both the mouse and object for the aged WT, tau−/− control, and tau−/− CypD mice. **(C)** Graph of the exploration time of both old and new mouse area for the aged WT, tau−/− control, and tau−/− CypD mice. **(D)** Graph of the familiarization phase illustrating the exploration time of both objects 1 and 2 for the aged WT, tau−/− control, and tau−/− CypD mice. **(E)** Heat maps of aged WT, tau−/− control, and tau−/− CypD groups during the NOR testing phase. **(F)** Graph of the exploration time of both the old and novel objects by the aged WT, tau−/− control, and tau−/− CypD mice, during the testing phase. Escape latency during training days 1 **(G)** and 2 **(H)** for the aged WT, tau−/− control, and tau−/− CypD mice. **(I)** Heat maps and representative tracks of aged WT, tau−/− control, and tau−/− CypD mice while seeking the escape chamber. Graph of the time in the quadrant **(J)** and the area **(K)** that the aged WT, tau−/− control, and tau−/− CypD mice spent to find the escape chamber. *p < 0.05, **p < 0.01, ***p < 0.001; mean ± S.E.M.

Considering that mitochondrial dysfunction can induce cognitive impairment (Mancuso et al., 2009) and that CypD overexpression in the aged tau−/− mice resulted in a loss of mitochondrial functionality, we sought to evaluate the behavioral performance of the tau−/− CypD mice. First, we evaluated their social capacity using the social interaction task (Jara et al., 2018). Figure 5A illustrates the heat maps that represent the behavior of the aged WT, tau−/−, and tau−/− CypD mice during the first stage. The aged tau−/− CypD mice explored for a shorter time than the aged tau−/− control mice, similar to the aged WT mice, showing similar exploration times for both the mouse and the object (Figure 5B). In the second stage, when the object was replaced by a new mouse, we observed that only the aged tau−/− mice spent more time investigating the new mouse (Figure 5C). In contrast, the aged WT and tau−/− CypD mice spent a similar time exploring the new and old mice (Figure 5C). These results indicated that overexpression of CypD in tau−/− mice reduced the social abilities that the aged tau−/− normally maintain during aging. Thus, the impairment of social behavior observed during aging could be related to the changes in CypD expression. Additionally, we subjected the mice to the NOR test. During the familiarization phase (Figure 5D), all groups of mice spent a similar amount of time exploring the two objects. In contrast, during the recognition phase, as shown in the heat maps, the behaviors of tau−/− mice and tau−/− CypD were different (Figure 5E). The aged tau−/− mice explored the novel object for a significantly longer time, whereas the tau−/− CypD mice spent the same amount of time as the WT mice in investigating the new object (Figure 5F). This indicated that aged tau−/− CypD mice were incapable of recognizing the old object, and therefore, the overexpression of CypD led to the loss of the recognition memory. Finally, we evaluated the effects of CypD overexpression on spatial learning and memory using the Barnes maze test (Figures 5G–I). On each day of training, we measured the escape latency, i.e., the time each mouse spent to find the escape chamber (Figures 5G,H). Our results showed that during the first training day, the aged tau−/− CypD mice and the aged tau−/− mice learned the location of the escape chamber at a similar time. This was in contrast to the aged WT mice that were incapable of finding the escape chamber (Figure 5G). During the second training day, we observed that the aged tau−/− mice exhibited reduced escape latency compared with the aged WT and tau−/− CypD mice (Figure 5H). These results indicated that, although aged tau−/− CypD mice initially learned faster than the aged WT mice, they had a similar behavior at the end of the training. Finally, 2 days after training in the Barnes maze, a new trial was performed in the absence of the escape chamber (Figures 5J,K). The behaviors of the experimental groups are shown in the heat maps and the representative tracks in Figure 5I. We measured the time that the mice spent in the quadrant of the escape chamber, and interestingly, the aged tau−/− mice remembered the escape zone, in contrast to the WT and tau−/− CypD mice (Figure 5J). More specifically, when we measured the time the mice explored the area around the escape chamber, a similar result was observed; the aged tau−/− mice found the correct location, whereas the tau−/− CypD mice had lost this ability, similar to the WT mice (Figure 5K). Therefore, these results indicated that overexpression of CypD in the absence of tau induced a loss of spatial memory and led to overall impairment of the hippocampal-dependent memory during aging.

Altogether, our results indicated that CypD overexpression in the aged tau−/− mice triggers mitochondrial dysfunction and memory loss similar to the aged WT mice. Thus, these data strongly suggested that tau contributes to physiological aging by a CypD-dependent mechanism. This is relevant because this is the first study demonstrating that tau negatively affects mitochondrial functionality and cognition during aging and suggests a new target for the treatment of aging-associated alterations.

## Discussion

In the present study, we used an aged (18-month) homozygous tau-knockout (tau−/−) to identify the importance of tau in the cognitive and mitochondrial alterations observed during aging. We report that aged WT mice exhibit an impairment of memory and social abilities, accompanied by reduced ATP production and high mitochondrial calcium sensitivity in the hippocampus. We show for the first time that the absence of tau prevents cognitive impairment in aged mice and mitochondrial dysfunction, evidenced by increased ATP production and reduced sensibility to mitochondrial calcium stress. Most importantly, the enhanced mitochondrial calcium-buffering capacity could be related to the reduction in CypD expression, since CypD overexpression in tau−/− mice led to ATP deficiency and premature mitochondrial swelling, probably due to mPTP opening. Thus, our results suggest that tau contributes to the mitochondrial and cognitive impairment in the hippocampus observed during normal aging and eventually to the development of neurodegenerative diseases, such as AD.

Loss of specific cognitive abilities is common during aging and in neurodegenerative diseases, such as AD (Fjell et al., 2014; Jara et al., 2019). The hippocampus is crucial for learning and memory; however, several studies suggest that the function of the hippocampus diminishes with age (Bettio et al., 2017). Likewise, other processes, dependent on the communication with the hippocampus, including social capacity, are affected by aging (Charles and Carstensen, 2010). In this study, we performed a battery of cognitive tests and report that aged tau−/− mice maintain their learning capacity, memorize, and recognize objects and spatial locations, in contrast to aged WT mice that showed a significant reduction in these capacities. Besides, we observed normal sociability in the aged tau−/− mice, while the aged WT mice lost the ability to interact with an unknown new mouse. These results are consistent with a previous report using the same genetic background of tau KO mice by Dawson et al. (2001) (C57BL/6J background; 4- to 5-month-old males) showing that tau deletion prevents memory decline in adult mice exposed to chronic stress (Lopes et al., 2016). While our work provides evidence that tau deletion prevents cognitive impairment during aging, other studies using tauGFP knock-in/knock-out mice (Stock No: 029219 | tauGFP, Jackson Laboratory) or C57Bl6/SJL (F1) female mice (Stock No. 100012, Jackson Laboratory) injected with Adeno-associated virus (AAV) construct containing a shRNA to MAPT gene showed that tau ablation in the hippocampus causes learning and memory deficits (Biundo et al., 2018; Velazquez et al., 2018). These contradictory results can probably be explained by the diverse genetic background, the methodology used to generate the ablation of tau, and the age of the animals used in the study.

Oxidative damage is characteristic of senescence and can affect brain functions (Palomera-Avalos et al., 2017; Jara et al., 2019). Oxidative stress is one of the most studied hypotheses to explain aging and neurodegeneration (Barja, 2014; Jara et al., 2019). Increased oxidative stress in the aging hippocampus is the result of an imbalance between the production of oxidative molecules and the anti-oxidant defense, leading to increased levels of ROS species (Uttara et al., 2009; Huang et al., 2016; Jara et al., 2019). Here, we demonstrate that the loss of tau prevents the oxidative damage associated with the formation of 4-HNE adducts, possibly by reducing ROS formation or increasing anti-oxidant activity. Therefore, these results suggest that the tau protein contributes to oxidative damage during aging, damage that is exacerbated in AD brains.

Mitochondria are the main producers of ROS, a sub-product of the respiratory chain (Chistiakov et al., 2014). Alterations in mitochondrial function lead to increased ROS production (Navarro and Boveris, 2010). In contrast, improvement of mitochondrial function has a beneficial effect (Rottenberg and Hoek, 2017). Supporting this idea, our group has previously reported that reduced oxidative damage in the hippocampus of young tau−/− mice could be the result of improved mitochondrial performance (Jara et al., 2018). Therefore, we evaluated this possibility in aged mice. Interestingly, we detected significantly increased ATP production in the aged tau−/− mice, indicating that mitochondrial bioenergetics are maintained during aging in the absence of tau. This could explain, almost in part, the early mitochondrial dysfunction observed in AD (Wang et al., 2020).

Mitochondrial function is influenced by fusion and fission events (Chistiakov et al., 2014; Wang et al., 2020). We demonstrated that the loss of tau did not induce changes in either fusion or fission proteins. Similarly, differences in the mitochondrial mass could have repercussions for mitochondrial function (Wenz, 2011; Chistiakov et al., 2014). However, we detected similar mitochondrial mass in the aged tau−/− and WT mice, indicating that the improvement of mitochondrial ATP production detected in the absence of tau is independent of mitochondrial dynamics or mass. Another possibility is an inefficient OXPHOS process as a consequence of alterations in the expression and/or activity of OXPHOS complexes (Reinecke et al., 2009; Tatarkova et al., 2011). Our results indicate that mitochondrial membrane potential and OXPHOS proteins did not change in the absence of tau. Therefore, considering that aged tau−/− mice present similar mitochondrial potential levels and expression of the OXPHOS complexes, higher ATP production may result from increased respiratory chain activity ATP synthase dependent. Future studies are needed to explore this possibility.

Mitochondria have additional functions to ATP formation (Perez et al., 2018a; Jara et al., 2019), contributing to cellular homeostasis and cell death (Hurst et al., 2017; Pérez and Quintanilla, 2017). These functions are partially regulated by the mPTP, a channel whose prolonged opening induces deterioration of cellular calcium homeostasis, oxidative stress, and decreased ATP production (Panel et al., 2018). Excessive production of ROS promotes mitochondrial and cellular oxidative damage, and neurodegeneration (Wenz, 2011; Jara et al., 2019). mPTP opening can be triggered by ROS and mitochondrial calcium overload (Hurst et al., 2017) both of which are enhanced in aging and age-related neurodegenerative diseases (Panel et al., 2018). The most known mPTP components are CypD, adenine nucleotide translocase (ANT), and ATP synthase (Rottenberg and Hoek, 2017; Panel et al., 2018), which mediate its function, although currently, its composition and structure, except for CypD, is not completely resolved (Jonas et al., 2015; Panel et al., 2018). We evaluated the components of this multi-protein complex, and interestingly, we observed that aged tau−/− mice had reduced levels of CypD, whereas the levels of the other proteins were similar to the levels seen in the aged WT mice. This is important because CypD is a crucial component for the formation of mPTP (Hom et al., 2011; Gauba et al., 2017; Perez and Quintanilla, 2017). In fact, our results are in concordance with previous studies showing that CypD deficiency increases mitochondrial function and cognitive abilities in transgenic mouse model of AD, indicating a protective effect in mice with neurodegenerative diseases (Du et al., 2011).

Mitochondria act by buffering high calcium concentrations (Hurst et al., 2017). However, when mitochondria are incapable of regulating calcium overload, they undergo swelling and promote mPTP opening, ultimately resulting in cell death (Perez and Quintanilla, 2017). To evaluate if reduced levels of CypD observed in tau−/− mice decreases the calcium sensibility that leads to mitochondrial swelling, we exposed isolated mitochondria to increasing calcium concentrations (Figure 4). Notably, we observed that the absence of tau reduced calcium sensitivity associated with mPTP opening. This is important because mitochondrial calcium dysregulation leads to prolonged mPTP opening and contributes to neurodegeneration, such as AD (Gauba et al., 2017; Panel et al., 2018). Therefore, we propose a possible role for tau in promoting mitochondrial mPTP-related swelling under physiological conditions, such as aging and neuronal damage that could lead to AD.

To validate that increased CypD levels induced by tau are responsible for mitochondrial and cognitive abnormalities detected in aged WT mice, we overexpressed CypD in aged tau−/− mice using a lentiviral vector.

Three weeks after transduction, we detected reduced mitochondrial bioenergetics in these animals, similar to that observed in aged WT mice. In particular, aged tau−/− mice overexpressing CypD presented reduced levels of ATP, accompanied by increased calcium sensitivity, associated with mPTP opening. These results indicate that the absence of tau contributes to improved mitochondrial function reducing CypD expression. Considering that mitochondrial function is fundamental for brain function and cognition gave the high energy demand of the synapses (Hara et al., 2014), this could explain the behavioral changes observed in WT mice. We observed negative behavioral changes, suggesting that overexpression of CypD affects recognition and spatial memory, and social abilities in the aged tau−/− mice. It is important to mention that we perform sham surgery in the control group, to reduce the effects of the surgical procedure when we compare these animals with tau−/− mice infected with a lentiviral vector containing CypD-GFP. However, to be sure that the complete effect is related to CypD overexpression and not to the expression of an unrelated gene, such as GFP, a lentiviral vector containing only GFP could be used; nevertheless, we do not have that viral vector at this time. Future studies may revise this question to discard effects related to GFP expression. Also, to confirm the dependence between tau and CypD on mitochondrial and hippocampal function during aging, a knockdown of CypD in aged WT mice will be generated, hoping that this will reduce age-associated cognitive and mitochondrial disturbances.

**FIGURE 6:**
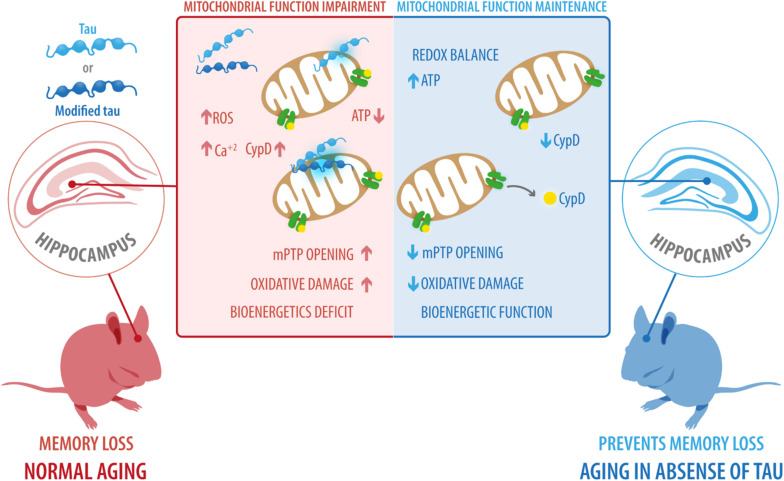
Possible role of tau protein on mitochondrial dysfunction and cognitive impairment during aging. Our results revealed that tau ablation significantly decreased the expression of CypD and prevented the mitochondrial and cognitive impairment associated with normal aging. However, based on studies in Alzheimer’s disease and other pathologies, we suggest two scenarios: (i) Modified forms of tau, including phosphorylated and cleaved forms, could induce increased ROS production and higher calcium concentrations, ultimately leading to mitochondrial dysfunction and mPTP opening; (ii) tau interact with mitochondrial proteins, such as CypD, promoting mitochondrial failure and more active mPTP. All these events could contribute to the cognitive deficits observed during normal aging. CypD, cyclophilin-D; mPTP, mitochondrial permeability transition pore; ROS, reactive oxygen species.

Our results revealed that tau ablation significantly decreased the expression of CypD and thus prevented the mitochondrial and cognitive impairment associated with normal aging. In light of these findings, additional studies are needed to understand the mechanism by which tau induces increased levels of CypD and mitochondrial dysfunction. However, based on studies on AD or other pathologies, two hypotheses have been proposed: (i) Modified forms of tau could induce increased ROS production and higher calcium concentrations (Panel et al., 2018; Perez et al., 2018a), which could result in mitochondrial dysfunction and ultimately in mPTP opening; (ii) Tau may interact with mitochondrial proteins (Liu et al., 2016), such as CypD (Amadoro et al., 2010) promoting mitochondrial failure and a more active mPTP. All these events could contribute to cognitive deficits observed in normal aging (Figure 6). The hypothesis that tau could interact with CypD are results of previous reports using human AD brain samples in which it was demonstrated that NH_2_-derived tau fragment interacts with CypD (Amadoro et al., 2012). This idea is also supported by previous studies of co-immunoprecipitation that indicate that tau interacts with an ample variety of proteins, of which 51% correspond to membrane-bound proteins (Liu et al., 2016). More specifically, within the membrane-associated target, 40.4% correspond to mitochondrial proteins (Liu et al., 2016).

## Data Availability Statement

The raw data supporting the conclusions of this article will be made available by the authors, without undue reservation, to any qualified researcher.

## Ethics Statement

Experimental procedures were approved by the Bioethical and Biosafety Committee of the Universidad Autónoma de Chile and Universidad San Sebastián, Santiago, Chile.

## Author Contributions

CJ, WC, CT-R, and RQ conceived the study. CJ and CT-R performed all experiments, analyzed the data, and wrote the manuscript. RQ and CT-R edited and prepared the final version of the paper. All authors read and approved the final version.

## Conflict of Interest

The authors declare that the research was conducted in the absence of any commercial or financial relationships that could be construed as a potential conflict of interest.

## Supplementary Material

Supplementary Figure 1 Mitochondrial mass in aged WT and tau−/− mice. **(A)** Representative images of unfixed hippocampal slices from WT and tau−/− mice stained with MitoTracker Green FM. **(B)** Quantitative analysis of MitoGreen staining in the DG, CA1, and CA3 hippocampal regions. **(C)** Western blot of hippocampal lysates and densitometric analysis of proteins involved in mitochondrial fusion, including Mfn1, Mfn2, and OPA1. **(D)** Western blot of hippocampal lysates and densitometric analysis of the proteins involved in mitochondrial fission, including phospho-Drp1, and total Drp1.
