# Supplementary figures and images for: Tau Deletion Prevents Cognitive Impairment and Mitochondrial Dysfunction Age Associated by a Mechanism Dependent on Cyclophilin-D
Source: Front Neurosci. 2021 Feb 10;14:586710. doi: 10.3389/fnins.2020.586710 (PMC7928299; doi:10.3389/fnins.2020.586710)

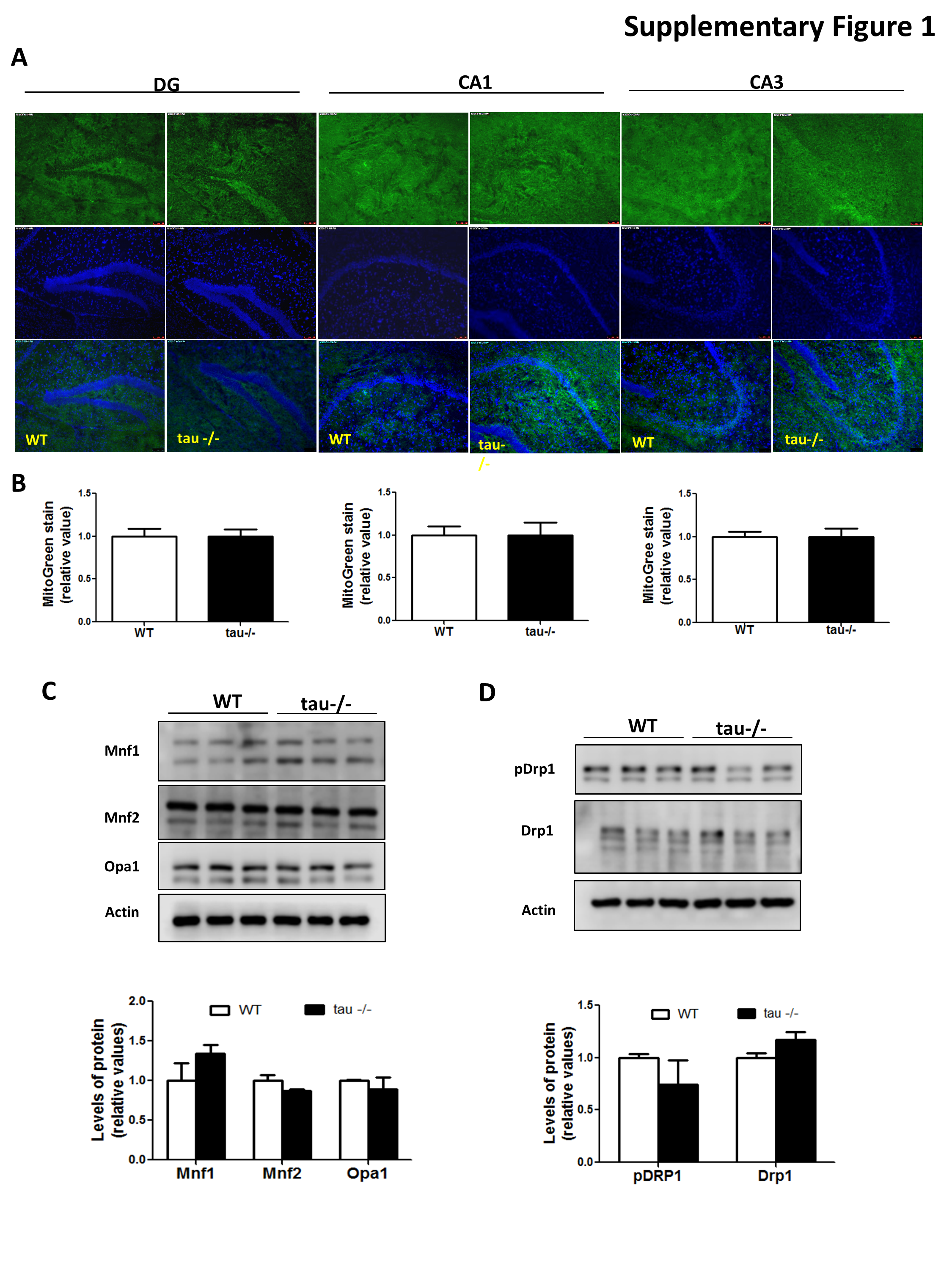

Supplement: Supplementary Figure 1 — Mitochondrial mass in aged WT and tau−/− mice. (A) Representative images of unfixed hippocampal slices from WT and tau−/− mice stained with MitoTracker Green FM. (B) Quantitative analysis of MitoGreen staining in the DG, CA1, and CA3 hippocampal regions. (C) Western blot of hippocampal lysates and densitometric analysis of proteins involved in mitochondrial fusion, including Mfn1, Mfn2, and OPA1. (D) Western blot of hippocampal lysates and densitometric analysis of the proteins involved in mitochondrial fission, including phospho-Drp1, and total Drp1. [file Image_1.TIF]
